# Hearing Loss and Urinary *trans*,*trans*-Muconic Acid (*t*,*t*-MA) in 6- to 19-Year-Old Participants of NHANES 2017–March 2020

**DOI:** 10.3390/toxics12030191

**Published:** 2024-02-29

**Authors:** Rae T. Benedict, Franco Scinicariello, Henry G. Abadin, Gregory M. Zarus, Roberta Attanasio

**Affiliations:** 1 Office of Innovation and Analytics, Agency for Toxic Substances and Disease Registry (ATSDR), Atlanta, GA 30341, USA; FScinicariello@cdc.gov (F.S.); HAbadin@cdc.gov (H.G.A.); GZarus@cdc.gov (G.M.Z.); 2 Department of Biology, Georgia State University, Atlanta, GA 30303, USA; rattanasio@gsu.edu

**Keywords:** children, benzene, hearing loss, adolescents, NHANES, pure tone audiometry

## Abstract

Hearing loss (HL) is associated with poorer language development and school performance. Ototoxic substances such as metals and solvents, including benzene, are a risk factor associated with HL. This study examines potential associations between the benzene metabolite *trans*,*trans*-muconic acid (*t*,*t*-MA) and HL in youth of the National Health and Nutrition Examination Survey (NHANES). Logistic regression calculated adjusted odds ratio (aOR) associations between HL and urinary *t*,*t*-MA quartiles, natural-log transformed, and doubled urinary *t*,*t*-MA. Hearing threshold pure-tone average (PTA) at speech frequencies (SF) 0.5, 1, 2, and 4 kHz and high frequencies (HF) 3, 4, and 6 kHz were analyzed for slight HL (PTA > 15 dB) and mild HL (PTA > 20 dB). Urinary *t*,*t*-MA was statistically significantly associated with both slight SF and HF HL. For each doubling of *t*,*t*-MA there were increased odds of having slight SFHL (aOR = 1.42; 95% CI: 1.05, 1.92), slight HFHL (aOR = 1.31; 95% CI: 1.03, 1.66), mild SFHL (aOR = 1.60; 95% CI: 1.10, 2.32), and mild HFHL (aOR = 1.45; 95% CI: 1.03, 2.04). To our knowledge, this is the first population-based report of an association between SFHL, HFHL, and the benzene metabolite *t*,*t*-MA in youth 6 to 19 years old.

## 1. Introduction

Hearing loss (HL) is a common chronic condition [1]. The Global Burden of Disease Study (2019) estimated that 1.57 billion people, or one in five people worldwide had hearing loss, of which 70 million were children aged 0–15 years [2]. Hearing impairment affects several aspects of the life of an individual, from communication to cognition, from employment to social activities and mental health [3]. Children with hearing loss have substandard language development and school performance, as well as behavioral problems [4,5,6]. Several factors are responsible for hearing loss in children, among them genetics, otitis media, childhood infections, noise exposure, ototoxic medicine, and ototoxic chemicals.

Although exposure to ototoxic substances such as aromatic solvents, halogenated hydrocarbons, and metals occur mostly in occupational settings, hearing impairments in children and adolescents have been associated with environmental exposure to lead [7,8], polycyclic aromatic hydrocarbons [9], and smoking or secondhand smoke exposure [10,11]. Exposure to aromatic solvents such as toluene, ethylbenzene, and benzene have been associated with hearing loss in adult participants of the National Health and Nutrition Examination Survey, NHANES 1999–2004 [12] and in petroleum refinery workers [13,14].

Exposure to benzene occurs primarily by inhalation, but skin and oral absorption may also occur. The general population may be exposed to benzene from gasoline fumes, automobile exhaust, emissions from some factories, and wastewater from certain industries. Benzene is commonly found in urban and rural air, with levels usually very low. However, exposures may be higher for people dwelling in enclosed spaces with unventilated fumes from gasoline, glues, solvents, paints, and art supplies. Additionally, gas stations and areas with heavy traffic and near industrial sources may have higher benzene air levels. Cigarette smoking and secondhand smoke are also important sources of exposure to benzene. People may also be exposed to benzene in contaminated drinking water and some foods [15]. Furthermore, a recent study indicated that an important indoor source of benzene is from the combustion of gas in (gas) stoves and ovens, with benzene levels higher than secondhand smoke [16]. Benzene may also be present in consumer products like cosmetics and personal care items [17].

The toxicity of benzene is caused by its metabolites, and among the urinary metabolites, *trans*,*trans*-muconic acid (*t*,*t*-MA) and S-phenylmercapturic acid (SPMA) have been commonly used as biomarkers of benzene exposure, both in occupational and environmental studies [18,19,20]. Due to the scarcity of information pertaining to the ototoxic effect of benzene in children and adolescents, we investigated whether there are associations between hearing loss and the urinary metabolite of benzene (*t*,*t*-MA) in NHANES participants aged 6 to 19 years old prior to the COVID-19 pandemic (2017–March 2020). The primary objective was to assess the potential association of at least slight hearing loss, both SFHL and HFHL, as well as any HL (SFHL and/or HFHL), with benzene exposure. Secondarily, we assessed the potential association of benzene exposure with mild hearing loss.

## 2. Materials and Methods

### 2.1. Study Population

The Centers for Disease Control and Prevention (CDC) National Center for Health Statistics conducts the NHANES, a nationally representative survey. This cross-sectional survey is of the United States non-institutionalized civilian population. The survey began in 1999 and has been conducted continuously with data released in 2-year cycles. Because of the COVID-19 pandemic, the NHANES 2019–2020 field operations were suspended in March 2020 and the data collected in 18 of the 30 survey locations were not nationally representative. Therefore, the data collected from 2019 to March 2020 were combined with data from the NHANES 2017–2018 cycle to form a nationally representative sample of NHANES 2017–March 2020 pre-pandemic data [21]. The NCHS Research Ethics Review Board approved the NHANES 2017–2018 and NHANES 2019–2020 study protocols [22] and all participants provided written informed consent. The study population included 6 to 19 year olds that participated in audiologic exams (otoscopy, middle ear testing, or audiometry), that had available urine measurements for *t*,*t*-MA and creatinine, and information regarding the covariates used in primary analyses (*n* = 686). Further, availability of serum cotinine measurements used in sensitivity analyses reduced the sample size to *n* = 590.

### 2.2. Audiometric Measurements

Trained examiners obtained audiometric measurements in a specifically designed and equipped audiometry room with a sound booth using a standardized protocol [23,24]. As described in Scinicariello et al. [11], air conduction thresholds were measured for each ear at 0.5, 1, 2, 3, 4, 6, and 8 kHz. Each ear was tested twice at the 1-kHz frequency and the average response used in the analyses. A difference of 10 dB or greater in the 1-kHz test–retest thresholds was deemed unacceptable, and these pure-tone audiograms were not used in the analyses.

### 2.3. Definition of Hearing Loss

Like other studies using NHANES, we used the average of four audiometric frequencies at 0.5, 1, 2, and 4 kHz to define the speech frequency (SF) PTA [25,26] and the average of the three audiometric frequencies at 3, 4, and 6 kHz to define a high frequency (HF) PTA [25,26,27]. We included only participants that had otoscopic screening exams and a priori we excluded those that had excessive or impacted ear cerumen (wax) (*n* = 5), collapsing external ear canals that were not normal (*n* = 0), ear compliance ≤ 0.2mL (*n* = 3), or pressure lower than −150 dekapascals (daPa) (*n* = 11). Ear compliance and pressure measure the ability of the middle ear to relay sound. Ear pressure below −150 daPa does not respond to sound efficiently. Ear compliance measures less than or equal to 0.2 mL indicate there is ossicular fixation or fluid in the ears. Slight hearing loss was defined as a PTA of 16 dB HL or greater in either ear and was used as a cutoff threshold to define slight hearing loss for both speech frequency and high frequency [28]. The World Health Organization’s (WHO) Global Burden of Disease Study [2] defines mild hearing loss as PTA > 20 dB, so we did the same.

### 2.4. Exposure Measurements

The concentrations of urinary metabolites of benzene were determined using ultra-performance liquid chromatography coupled with electrospray ionization tandem mass spectrometry (UPLC–ESI-MS/MS) [29] by the Division of Laboratory Sciences (DLS), National Center for Environmental Health (NCEH), CDC. Two metabolites of benzene, *trans*,*trans*-muconic acid (*t*,*t*-MA) and phenylmercapturic acid (PMA), were measured in participants aged 6–19 years from a one-third subsample of the NHANES 2017–March 2020 sample. Due to the high percentage (66.1%) of PMA below the detection limit (LLOD = 0.150 ng/mL), in our sample we used only measurements of urinary *t*,*t*-MA where more than 92% were at or above the limit of detection (LLOD = 9.81 ng/mL). Spot urinary samples may be diluted or concentrated depending on the physical health of the participant and the amount of liquid intake. To account for variation in dilution of spot urinary samples, we adjusted for urinary creatinine by including it as a model covariate [30].

### 2.5. Sociodemographic and Hearing-Related Variables

Sociodemographic and hearing-related variables that influence or are suspected of influencing hearing thresholds were used in the . Like Scinicariello et al. [11], the variables used for analyses were age, sex, racial and ethnic group, poverty income ratio (PIR), body weight status (normal/underweight, overweight, and obese), and self-reported ear infection. Additionally, survey responses for “exposed to very loud noise or music for 10 or more hours a week for a period of 3 months or longer” or “ever been exposed to very loud noise or music for 10 or more hours a week” were added to the analyses. In sensitivity analyses, we used serum cotinine as a biomarker of exposure to both environmental smoke and/or active tobacco use to determine whether smoking could confound the urinary *t*,*t*-MA results. We categorized people from racial and ethnic groups as non-Hispanic White, non-Hispanic Black, Hispanic, and Other (Asian-American, Other Race, and Multi-race).

Dividing the weight by height squared (kg/m^2^) calculated the body mass index (BMI). The relation between BMI and body weight in children depends on age and sex, therefore obesity was defined as a BMI at or above the 95th percentile of the CDC sex-specific BMI-for-age growth charts [31]. A BMI between the 85th and 95th percentiles was considered overweight. A BMI less than the 5th percentile was defined as underweight. A BMI between the 5th percentile to less than the 85th percentile was considered normal weight. Because of the small sample size of underweight participants with hearing impairment, they were combined with normal weight individuals.

The household interview gathered information about self-reported ear infection and noise or music exposure. Generally, persons 16 years of age and older and emancipated minors were interviewed directly. A responsible adult provided information for participants under 16 years of age.

### 2.6. Statistical Methods

All analyses were performed using the urinary benzene metabolite (*t*,*t*-MA), specific sample weights for the subsample, WTVOC2PP, as recommended by NCHS, and to account for the complex sampling design and non-response of NHANES [21]. Statistical analyses used SAS 9.4 (SAS Institute, Cary, NC, USA) and SAS-Callable SUDAAN 10 (Research Triangle Institute, Research Triangle Park, NC, USA).

Logistic regressions calculated odds ratios (ORs) or adjusted ORs (aORs) and 95% CIs for hearing loss outcomes. Wald statistical tests for linear trends were conducted by modeling the ordinal variable using integer values. Weighted quartiles were established based on the weighted distributions in the study population.

We ran two models using the covariates: Model 1 included age, sex, racial and ethnic group, obesity, PIR, ear infections, loud noise exposure, and urinary creatinine; Model 2, are sensitivity analyses that included Model 1 covariates and serum cotinine. To investigate the associations and the shape relationship between the outcomes and the chemical, *t*,*t*-MA was modelled: (1) as a natural log-transformed (ln) continuous variable, and (2) as a categorical variable according to quartile, based on the weighted distribution of the chemical in the study population.

Geometric means were used to assess all biomonitoring data fields, as they provide better estimates of central tendency when data have long tails in the distribution [32,33]. Moreover, we estimated aORs for hearing loss associated with a twofold increase of *t*,*t*-MA concentration by exponentiating the coefficient of the log of the aOR.

The log of odds ratio Y was calculated using the formula Y = X/Log2 (e), where X is the coefficient for the log of odds ratio from logistic regression when the natural logarithm of *t*,*t*-MA is used. Log2 (e) is the logarithm in base 2 of the Euler numerical constant (e = 2.71828). Exponentiating the coefficient Y will estimate the odds ratio of the hearing loss associated with a twofold increase *t*,*t*-MA concentration.

## 3. Results

Table 1 shows the characteristics of the study population (*n* = 686). The geometric mean (GM) age of all participants was 12.6 years. Among the participants, 50% were female, 52.5% were non-Hispanic White, 18.1% were obese, and 27.7% were from families with income below the poverty level. Seven percent reported exposure to loud noise, and 59.3% of the participants reported three or more ear infections. The geometric mean of *t*,*t*-MA was 66.07 ng/mL (range min= 6.94 ng/mL; max = 8640 ng/mL). The weighted prevalence of at least slight SFHL > 15 dB and HFHL > 15 dB was 5.8% (48 cases and 636 controls) and 9.1% (11 cases and 604 controls), respectively. Whereas the weighted prevalence of at least mild SFHL > 20dB and HFHL > 20 dB was 2.6% (20 cases and 664 controls) and 4.0% (31 cases and 650 controls) respectively (Table 2).

Supplement Table S1 presents the logistic regression results of the crude analyses between slight hearing loss outcomes and chemical concentrations. Table 3 presents the results of multivariable logistic regression analyses. There were statistically significant associations between the urinary benzene metabolite *t*,*t*-MA with slight SFHL and HFHL, as well as for any HL (Table 3, Model 1). Further adjustments with serum cotinine indicated that SFHL and HFHL were statistically significantly associated with each unit increase of natural log-transformed *t*,*t*-MA (aOR = 1.66; 95% CI: 1.07–2.57, and aOR = 1.47; 95% CI: 1.04–2.09, respectively) (Table 3, Model 2). For each doubling of urinary *t*,*t*-MA there were 42%, 31%, and 32% increase in odds of having SFHL, HFHL, or any HL (Table 3, Model 2).

Analyses by chemical quartiles showed that the highest quartile *t*,*t*-MA, compared to the referent quartile was associated with increased adjusted odds of having slight SFHL (aOR = 4.02; 95% CI: 1.04–15.56), slight HFHL (aOR = 4.00; 95% CI: 1.02–15.75), and any slight HL (aOR = 3.54; 95% CI: 1.03–12.17) (Table 3, Model 2). There was evidence of dose–response relationships based on statistical significance of the *p*-values for the trend (*p* < 0.05) in all models (Table 3).

Further, we analyzed multivariate models using the definition of mild hearing loss at PTA > 20 dB for children and adolescents [2]. Because of the smaller sample size of cases, mild HL outcomes were modelled using continuous log-transformed urinary *t*,*t*-MA. Table 4 shows that increased levels of *t*,*t*-MA were associated with mild SFHL (SFHL > 20 dB), mild HFHL (HFHL > 20 dB), and any mild HL. For each doubling of *t*,*t*-MA there were 60% (17 cases and 572 controls), 45% (28 cases and 557 controls), and 47% (31 cases and 559 controls) higher odds of mild hearing loss at speech frequency, high frequency, and any mild HL, respectively (Table 4, Model 2). Supplement Table S2 shows the results also using *t*,*t*-MA quartiles. The absence of cases in the third urinary *t*,*t*-MA quartile resulted in no available aOR.

## 4. Discussion

Children and youth with hearing impairment face multiple challenges, from speech and communication problems to psychological and behavioral problems [34]. Children with minimal hearing loss compared to their normal hearing peers have more academic difficulties and are more likely to fall behind a grade year [35]. Children with hearing impairment have lower self-esteem in perceived social acceptance by their peers [36] and they are more prone to depressive symptoms than their normal hearing peers [37].

In our analyses we found that urinary *t*,*t*-MA, the biomarker of exposure to benzene, was positively associated with increased odds of both slight, as well as mild speech frequency and high frequency hearing loss in individuals 6 to 19 years of age. To our knowledge, this is the first population-based report of an association between HL and a biomarker of benzene exposure in this age group. Slight HL was observed to be significant above a *t*,*t*-MA value of 156.50 ng/mL (Table 3). Previously, Staudt et al. [12], using NHANES cycles 1999–2004, reported a positive association between blood benzene and higher odds of having high-frequency hearing loss (≥25 dB) in adults 20 to 59 years old. In occupational settings, benzene, as part of chemical mixture exposures, has been associated with hearing loss [14,38,39].

The common key event of the diverse risk factors (e.g., noise, smoking, toxic substances, diabetes) in developing hearing loss is via the production of reactive oxygen species, ROS [13,40,41]. Excessive production of ROS species may induce mitochondrial DNA mutations with resulting cochlear cell apoptosis [41]. The production of ROS and oxidative stress may be a plausible link in the relationship that we found between *t*,*t*-MA and HL. Increased oxidative stress markers and increased risk of insulin resistance with increased urinary *t*,*t*-MA have been reported in children [42] and in adults [43]. In an occupational setting, benzene exposure and urinary *t*,*t*-MA were associated with increased oxidative protein damage, decreased antioxidant capacity, and decreased glutathione activity [44].

Tobacco smoke has been associated with increased risk of hearing loss [45,46]. Tobacco smoke comprises a complex blend of chemicals generated through the combustion of tobacco and its additives. Among the generated chemicals is benzene and studies in adults have shown higher urinary *t*,*t*-MA in smokers vs. non-smokers [47,48,49]. In our sensitivity analyses, when we adjusted for serum cotinine as a biomarker of exposure to tobacco smoke, there was no relative change in the magnitude of the *t*,*t*-MA associated with hearing loss, suggesting that smoke exposure was not a confounder in this study.

Although *t*,*t*-MA is a widely used biomarker of exposure to benzene, there are concerns about the use of urinary *t*,*t*-MA as a biomarker at low levels of benzene exposure because of a lack of specificity [47,50,51,52]. Urinary *t*,*t*-MA is also a metabolite of the food preservative sorbic acid [15]; therefore, the attributable contribution of urinary *t*,*t*-MA derived from benzene exposure in our population may be confounded by sorbic acid present in dietary food. However, we must also consider that benzene has been reported as present in food and beverages. Benzene is formed in beverages containing both benzoate salts and ascorbic acid [53,54]. Furthermore, benzene concentrations from gas stove and oven combustion can be more hazardous than the concentration from secondhand smoke [16]. It is plausible that children are being exposed to benzene in homes with gas combustion appliances since Kasthan et al. [16] observed that benzene can migrate into other rooms far from the kitchen. Indeed, measurements of benzene in bedrooms exceeded chronic health benchmarks for several hours even after the stove was turned off [16].

There are several other limitations in our study. A major study limitation is the measurement of audiometric thresholds at a single point in time as the identified audiometric notches may only represent temporary threshold shifts. The cross-sectional nature of this study limits the inferences that can be made based on the results. As a cross-sectional study, where measures are taken simultaneously, it is not possible to determine whether the exposure preceded hearing loss. The short half-life of benzene and use of a single spot urine sample also limits our ability to make inferences. The associations reported in this study could also be biased by uncontrolled factors such as genetic predisposition, otosclerosis, diabetes, ototoxic medication, and exposure to other ototoxic substances [3].

## 5. Conclusions

The data analyses indicate there are significant associations of *t*,*t*-MA with several hearing loss outcomes in children and adolescents at both speech and high frequency levels. These findings may provide valuable information about exposure and potential health risks. Youth with hearing impairment have been shown to experience academic difficulties, behavioral problems, and lower performance in oral language compared to their peers with normal hearing. Since hearing impairment progresses insidiously for years before being self-perceived or diagnosed, routine hearing screenings may provide early diagnosis, opportunities for noise prevention counseling, and the ability to undertake early preventive measures [3]. People can take measures to reduce their benzene exposure by avoiding smoke (environmental or secondhand smoke) and reducing/removing gas appliances from inside homes. Since *t*,*t*-MA is also a by-product of the added food preservative sorbic acid, lower intake of processed food may also reduce formation of *t*,*t*-MA.

## Figures and Tables

**Table 1:** Weighted characteristics of participants (6–19 years of age) in NHANES 2017–March 2020 (*n* = 686).

Variable	All % or GM (SE)	Normal Hearing % or GM (SE)	Any Slight Hearing Loss > 15 dB % or GM (SE)
Age (years)	12.6 (0.18)	12.5 (0.21)	12.6 (0.55)
Male	50.0 (2.59)	50.7 (2.65)	42.0 (6.10)
Female	50.0 (2.59)	49.3 (2.65)	58.0 (6.10)
*trans*,*trans*-muconic acid (*t*,*t*-MA) (ng/mL)	66.07 (3.97)	62.08 (4.81)	111.51 (23.73)
Urinary creatinine (mg/dL)	95.82 (3.94)	93.99 (4.39)	109.71 (7.79)
Serum Cotinine ^†^ (ng/mL)	0.07 (0.01)	0.06 (0.01)	0.22 (0.09)
			
Underweight/normal (BMI < 85th percentile)	62.0 (2.18)	63.8 (2.40)	45.9 (9.62)
Overweight (BMI 85th < 95th percentile)	19.9 (2.24)	19.4 (2.06)	24.5 (10.67)
Obese (BMI ≥ 95th percentile)	18.1 (1.97)	16.8 (2.23)	29.6 (7.59)
			
Non-Hispanic White	52.5 (3.60)	53.7 (3.6)	41.2 (6.84)
Non-Hispanic Black	12.9 (2.48)	11.9 (2.27)	21.2 (6.46)
Hispanic	22.8 (3.14)	22.3 (3.23)	27.6 (5.48)
Others and Multi-race	11.8 (1.58)	12.1 (1.70)	10.0 (3.23)
			
Household income PIR < 1.3	27.7 (2.49)	26.3 (2.49)	38.6 (7.97)
Household income PIR 1.3–4.99	52.6 (3.19)	53.2 (3.16)	49.2 (7.59)
Household income PIR = 5	19.7 (2.49)	20.6 92.67)	12.2 (5.54)
			
Had 3 or more ear infections	59.3 (3.43)	60.0 (3.34)	53.4 (6.37)
Exposed to loud noise, yes	7.0 (1.09)	6.8 (1.29)	6.9 (6.37)

BMI = body mass index; GM = geometric mean; SE = standard error; PIR = poverty income ratio. ^†^ Serum cotinine measurement available only in 590 participants.

**Table 2:** Weighted prevalence of speech frequency (SF) and high frequency (HF) HL in 6–19-year-old participants of NHANES 2017–March 2020 (*n* = 686).

	Speech Frequency HL	High Frequency HL	Any SFHL or HFHL
Slight HL > 15 dB			
*n* cases/*n* controls	48/636	77/604	89/597
% (SE)	5.8 (1.00)	9.1 (1.49)	10.5 (1.44)
Mild HL > 20 dB			
*n* cases/*n* controls	20/664	31/650	34/652
% (SE)	2.6 (0.87)	4.0 (0.87)	4.2 (0.90)

HF = high frequency; HL = hearing loss; SE = standard error; SF = speech frequency.

**Table 3:** Multivariate logistic regression adjusted ORs (95% CI) of slight hearing loss outcomes and urinary *t*,*t*-MA ^a^ levels for US participants aged 6- to 19-years, NHANES 2017–March 2020.

	Slight Speech Frequency Hearing Loss > 15 dB (SFHL)		Slight High FrequencyHearing Loss >15 dB (HFHL)		Any Slight Hearing Loss > 15 dB	
	Model 1	Model 2	Model 1	Model 2	Model 1	Model 2
Case/control, *n*	48/636	41/548	77/604	64/521	89/597	74/516
*t*,*t*-MA (natural log)	1.66 (1.12, 2.47) *	1.66 (1.07, 2.57) *	1.49 (1.10, 2.03) *	1.47 (1.04, 2.09) *	1.51 (1.10, 2.06) *	1.49 (1.04, 2.12) *
*t*,*t*-MA doubling	1.42 (1.08, 1.87) *	1.42 (1.05, 1.92) *	1.32 (1.07, 1.63) *	1.31 (1.03, 1.66) *	1.33 (1.07, 1.65) *	1.32 (1.03, 1.69) *

*t*,*t*-MA Quartiles (ng/mL)						
Q1: ≤29.17	1 (referent)	1 (referent)	1 (referent)	1 (referent)	1 (referent)	1 (referent)
Q2: 29.18-61.51	0.97 (0.24, 3.99)	0.70 (0.10, 5.05)	1.14 (0.31, 4.16)	1.24 (0.25, 6.16)	1.05 (0.35, 3.20)	0.96 (0.22, 4.11)
Q3: 61.52-156.50	0.78 (0.18, 3.44)	0.55 (0.13, 2.32)	1.81 (0.35, 9.22)	1.45 (0.26,8.07)	1.80 (0.48, 7.18)	1.35 (0.32, 5.67)
Q4: >156.50	4.11 (1.23, 13.73) *	4.02 (1.04, 15.56) *	3.94 (1.17, 13.21) *	4.00 (1.02, 15.75) *	3.74 (1.29, 10.87) *	3.54 (1.03, 12.17) *
*p*-trend	0.0047 *	0.0007 *	0.0016 *	0.0028 *	0.002 *	0.004 *

^a^ *t*,*t*-MA has been weighted using specific sample weights for the subsample, WTVOC2PP, as recommended by NCHS [21]. Model 1 adjusted for age, sex, racial and ethnic group, poverty income ratio (PIR), body weight (normal/underweight, overweight, and obese), self-reported ear infection, loud noise exposure, and urinary creatinine. Model 2 is Model 1 plus serum cotinine. * Indicates associations with statistical significance (*p* < 0.05).

**Table 4:** Multivariate logistic regression adjusted ORs (95% CI) of mild hearing loss outcomes and urinary *t*,*t*-MA ^a^ levels for US participants aged 6- to 19-years, NHANES 2017–March 2020.

	Mild Speech Frequency Hearing Loss > 20 dB (SFHL)		Mild High Frequency Hearing Loss > 20 dB (HFHL)		Any Mild Hearing Loss > 20 dB	
	Model 1	Model 2	Model 1	Model 2	Model 1	Model 2
Case/control, *n*	20/664	17/572	31/650	28/557	34/652	31/559
*t*,*t*-MA (natural log)	1.79 (1.03, 3.14) *	1.97 (1.15, 3.37) *	1.64 (1.00, 2.67) *	1.71 (1.04, 2.81) *	1.67 (1.04, 2.70) *	1.75 (1.06, 2.87) *

*t*,*t*-MA doubling	1.50 (1.02, 2.21) *	1.60 (1.10, 2.32) *	1.41 (1.00, 1.97) *	1.45 (1.03, 2.04) *	1.43 (1.03, 1.99) *	1.47 (1.04, 2.07) *

^a^ *t*,*t*-MA has been weighted using specific sample weights for the subsample, WTVOC2PP, as recommended by NCHS [21]. Model 1 adjusted for age, sex, racial and ethnic group, poverty income ratio (PIR), body weight (normal/underweight, overweight, and obese), self-reported ear infection, loud noise exposure, and urinary creatinine. Model 2 is Model 1 plus serum cotinine. * Indicates associations with statistical significance (*p* < 0.05).

## Data Availability

NHANES datasets may be obtained at https://wwwn.cdc.gov/Nchs/Nhanes/Search/default.aspx (accessed on 27 February 2024).

## Supplementary Materials

The following supporting information can be downloaded at https://www.mdpi.com/article/10.3390/toxics12030191/s1: Table S1: Logistic regression ORs (95% CI) of slight hearing loss outcomes and urinary *t*,*t*-MA^a^ levels for US participants 6–19 year-old participants of NHANES 2017–March 2020. Table S2: Multivariate logistic regression adjusted ORs (95% CI) of mild hearing loss outcomes and urinary *t*,*t*-MA^a^ quartiles for US participants 6–19 year-old participants of NHANES 2017–March 2020.
