# Supplementary material for: Hearing Loss and Urinary trans,trans-Muconic Acid (t,t-MA) in 6- to 19-Year-Old Participants of NHANES 2017–March 2020
Source: Toxics. 2024 Feb 29;12(3):191. doi: 10.3390/toxics12030191 (PMC10974904; doi:10.3390/toxics12030191)
Supplement: Supplementary file 1 [file toxics-12-00191-s001.zip › toxics-2875065-supplementary.pdf]

Hearing Loss and Urinary *trans,trans*-Muconic Acid (*t,t*-MA) in 6- to 19-Year-Old Participants of NHANES 2017–March 2020

Rae T. Benedict [1,*], Franco Scinicariello [1], Henry G. Abadin [1], Gregory M. Zarus [1] and Roberta Attanasio [2]

Table S1. Logistic regression ORs (95% CI) of slight hearing loss outcomes and urinary *t,t*-MA[a] levels for US participants 6–19 year-old participants of NHANES 2017–March 2020.

	**Slight Speech Frequency Hearing Loss >15dB (SFHL)**	**Slight High Frequency Hearing Loss >15dB (HFHL)**	**Any Slight Hearing Loss >15dB**
	Crude Model	Crude Model	Crude Model
Case/control, *n*	48/636	77/604	89/597
t,t-MA (natural log)	1.47 (1.01, 2.12)*	1.47 (1.09, 1.99)*	1.41 (1.05, 1.91)*
t,t-MA doubling	1.30 (1.01, 1.68)*	1.31 (1.06, 1.61)*	1.27 (1.03, 1.56)*
t,t-MA Quartiles (ng/mL)			
Q1: ≤ 29.17	1 (referent)	1 (referent)	1 (referent)
Q2: 29.18-61.51	0.76 (0.22, 2.61)	1.20 (0.39, 3.71)	0.94 (0.36, 2.46)
Q3: 61.52-156.50	0.65 (0.16, 2.62)	2.00 (0.44, 9.11)	1.74 (0.48, 6.37)
Q4: >156.50	3.08 (1.07, 8.87)*	4.11 (1.35, 12.53)*	3.25 (1.20, 8.80)*
p-trend	0.01*	0.0046*	0.007*

[a] *t,t*-MA has been weighted using specific sample weights for the subsample, WTVOC2PP, as recommended by NCHS [21].
Crude model does not adjust for any confounders. *Indicates associations with statistical significance (p<0.05).

Table S2. Multivariate logistic regression adjusted ORs (95% CI) of mild hearing loss outcomes and urinary *t,t*-MA[a] quartiles for US participants 6–19 year-old participants of NHANES 2017–March 2020.

	**Mild Speech Frequency Hearing Loss >20dB (SFHL)**		**Mild High Frequency Hearing Loss >20dB (HFHL)**		**Any Mild Hearing Loss >20dB**	
	Model 1	Model 2	Model 1	Model 2	Model 1	Model 2
Case/control, *n*	20/664	17/572	31/650	28/557	34/652	31/559
t,t-MA Quartiles (ng/mL)						
Q1: ≤ 29.17	1 (referent)	1 (referent)	1 (referent)	1 (referent)	1 (referent)	1 (referent)
Q2: 29.18-61.51	1.06 (0.12, 9.46)	1.29 (0.09, 18.93)	0.97 (0.20, 4.69)	1.16 (0.16, 8.38)	0.97 (0.20, 4.74)	1.10 (0.15, 8.14)
Q3: 61.52-156.50	0.14 (0.01, 1.63)	-[b]	0.19 (0.04, 0.96)	0.12 (0.01, 0.98)	0.20 (0.04, 1.00)	0.12 (0.01, 0.98)
Q4: >156.50	4.69 (0.75, 29.27)	7.75 (1.01, 59.66)	4.35 (1.13, 16.69)	5.93 (1.18. 29.75)	4.67 (1.24, 17.55)	6.27 (1.27, 31.04)
p-trend	0.008*	0.001*	0.0001*	0.0005*	0.005*	0.0004*

[a] *t,t*-MA has been weighted using specific sample weights for the subsample, WTVOC2PP, as recommended by NCHS [21]. Model 1 adjusted for age, sex, people from racial and ethnic groups, poverty income ratio (PIR), body weight status (normal/underweight, overweight, and obesity), self-reported ear infection, loud noise exposure and urinary creatinine. Model 2 is model 1 plus serum cotinine. [b]Adjusted OR was not possible because there are zero cases in the third quartile. *Indicates associations with statistical significance ($p<0.05$).
